# Clinical diagnostic value of the plasma heparin binding protein in diabetic nephropathy patients comorbid with sepsis

**DOI:** 10.12669/pjms.42.2.12815

**Published:** 2026-02

**Authors:** Lulu Han, Shenghai Wang, Zhao Wang

**Affiliations:** 1 Lulu Han Department of Endocrinology, Baoding No.1 Central Hospital, Baoding 071000, Hebei, China; 2 Shenghai Wang Intensive Care Unit, Affiliated Hospital of Hebei University, Baoding 071000, Hebei, China; 3 Zhao Wang Department of Endocrinology, Baoding No.1 Central Hospital, Baoding 071000, Hebei, China

**Keywords:** Diagnostic value, Diabetic nephropathy, Heparin binding protein, Sepsis

## Abstract

**Objective:**

To explore the clinical diagnostic value of plasma heparin binding protein in diabetes nephropathy (DN) with sepsis.

**Methodology:**

The study was a retrospective study of 82 patients with DN, carried out in Affiliated Hospital of Hebei University from April 2024 to December 2024. Information about inflammatory indicators, biochemical, glucose metabolism and urine microalbumin indicators. Patients were divided into two major groups, DN sepsis group (n=42) and DN group (n=40). Date of 40 T2DM patients were used as the normal control group. The inter group comparison was conducted using analysis of variance, Pearson correlation analysis was used to analyze the correlation between HBP and BUN, Scr, UACR, and eGFR, and receiver operating characteristic (ROC) curve analysis was used to evaluate the specificity and sensitivity of inflammatory indicators, and to evaluate their diagnostic efficiency for DN patients with sepsis.

**Results:**

Compared with DN and T2DM groups, HBP, CRP and PCT were dramatically increased in DN sepsis group (p<0.001), ESR and NE% also higher (p<0.05). In DN group, HBP exhibited an upward trend (p<0.05) than T2DM group. The serum HBP was positively correlated with BUN, Scr and UACR, negatively correlated with eGFR in DN patients. In the early stage of sepsis in DN patients, the area under the curve (AUC) of HBP is 0.691.

**Conclusion:**

The plasma HBP were dramatically increased in DN patients comorbid with sepsis, plasma HBP clinical testing were essential and had high-efficiency clinical diagnosis abilities of sepsis in DN patients.

## INTRODUCTION

Diabetes Nephropathy (DN) is a renal microcirculation disorder caused by chronic hyperglycemia and is one of the most serious complications of Type-II diabetes mellitus (T2DM). DN patients often have excessive activation and dysfunction of vascular endothelium, which can cause abnormalities in blood rheology and promote coagulation,^1^,^2^ especially in cases of severe infection. Therefore, when DN patients have sepsis, there is a greater risk of developing microcirculatory disorders and organ failure. Sepsis can cause septic shock and life-threatening organ failure. Its high incidence rate, high mortality and high economic costs are one of the major public health problems in the world.^3^

In DN patients, sepsis can induce excessive endothelial activation, accelerate microcirculatory disorders and exacerbate the original degree of kidney damage. Therefore, early identification and effective treatment of DN complicated with sepsis remain the main challenge. Heparin binding protein (HBP) is mainly derived from secretory granules and cyanophilic granules of polymorphonuclear leukocytes. Research has shown that HBP is involved in the regulation of inflammatory response and vascular leakage, exhibiting higher sensitivity in identifying locally infected sepsis and directly reflecting the progression of sepsis in patients. The increase of HBP may cause renal edema by increasing vascular permeability, glomerular filtration and impaired renal tubular function.^4^

At present, DN patients still lack sensitive and specific diagnostic markers in the early stages of infection. In this study, we evaluated the plasma HBP levels of DN patients with sepsis, exploring whether plasma HBP can serve as a sensitive and specific diagnostic marker for sepsis in DN patients and whether HBP levels can reflect the degree of renal injury in DN combined with sepsis.

## METHODOLOGY

This was a retrospective study of 82 patients with DN, carried out in Affiliated Hospital of Hebei University from April 2024 to December 2024, information about patients were collected through the hospital medical record system and retrospectively, among them 42 comorbid with sepsis and named as DN sepsis group; another 40 DN without any infectious diseases were named as DN group. Besides 40 T2DM without any complications named as T2DM group.

### Ethical approval:

The study was approved by the Institutional Ethics Committee of Affiliated Hospital of Hebei University (No: HDFYLL-KY-2023-153; Date: August 30, 2023) and written informed consent was obtained from all participants.

### Inclusion criteria:

- Age for patients limited to 18-80 years.
- Have the real medical information’s and complete clinical data.
- Patients or their legal guardians can understand all content of this research fully and completely, accept and signed a written informed consent.

### Exclusion criteria

- Patients with Type-1 or other types of diabetes;
- Patients with diabetes ketosis, ketoacidosis, hyperglycemia, hypertonic state;
- Patients with autoimmune system or taking immunosuppressants or nephrotoxic drugs;
- Patients without complete clinical data or giving up treatment.

The diagnosis of T2DM and DN were based on the guidelines of the American Diabetes Association in 2021.^5^,^6^ The definition of sepsis was biassed on the Third International Consensus Definitions for Sepsis and Septic Shock (Sepsis-3), the quick Sequential Organ Failure Score (qSOFA) or the Sequential Organ Failure Assessment (SOFA) criteria were used to diagnose sepsis patients.^7^

### Data collection:

HBP, C reactive protein (CRP) and procalcitonin (PCT) through dry immunofluorescence quantitative method, erythrocyte sedimentation rate (ESR) was tested by automatic sedimentation meter, white blood cell (WBC) and the percentage of neutrophil count (NE%) were measured by fully automatic blood body fluid analyzer. Simultaneous detection fasting blood glucose (FBG), serum creatinine (SCr), blood urea nitrogen (BUN), glycosylated hemoglobin (HbA1c), the eGFR should be calculated by using the Chronic Kidney Disease Epidemiology Collaboration (CKD-EPI) 2021 formula.^8^ Measure urine microalbumin (mALB) and creatinine (Cr) via immunoturbidimetry further calculated the UACR (mALB/ Cr).

### Statistical analysis:

Statistical Analysis were performed in GraphPad Prism 8.0 software (La Jolla, CA, USA). The normally distributed data information’s were expressed as means±standard deviation (SD). Categorical data were assessed using a Chi-square test. One-way ANOVA with the Tukey’s multiple comparisons test for significant difference among multiple groups with single factor. Pearson correlation analysis was used to analyses the correlation between two normally distributed parameters, receiver operating characteristic (ROC) curve analysis was used to assess the specificity and sensitivity of the inflammatory indicators and evaluated their diagnostic efficiency on DN patients with sepsis. Results were considered significant at p< 0.05.

## RESULTS

The general data of three group patients is showed in Table-I. The FBG was obviously higher in DN with sepsis than the other two groups(p<0.05). The eGFR in DN group were lower and UACR were obviously raised than T2DM patients (p<0.05). The renal function in DN sepsis group was aggravated, UACR BUN, Scr were remarkably increased and eGFR were progressively declined than the other two groups (all p<0.05).

**Table-I:** Demographic characteristics and general indicators of patients in three groups.

Demographic characteristics and General indicators	T2DM group	DN group	DN sepsis group	F(X^2^)	p
Age (years)	48.38±12.38	47.73±15.45	54.14±13.35	2.72	0.07*
Gender (male/female)	18/22	23/17	22/20	1.27	0.53^#^
BMI (kg/m^2^)	23.05±3.38	24.35±4.19	25.18±2.14	4.25	0.16*
FBG (mmol/L)	9.03±6.14	9.77±2.53	11.36±3.09	3.31	0.04*
HbA1c (%)	7.75±1.84	8.42±1.63	8.67±1.97	2.77	0.07*
BUN (mmol/L)	4.05±1.36	5.93±2.14	8.95±2.55	57.70	<0.001*
Scr (umol/L)	58.48±13.97	79.76±23.10	119.6±102.10	10.34	<0.001*
eGFR (mL/min/1.73m^2^)	114.30±12.84	92.95±23.18	76.43±32.96	24.39	<0.001*
UACR (mg/g)	8.95±6.76	241.70±266.60	510.70±351.00	39.34	<0.001*

* One-way ANOVA,

# Chi-square test, p< 0.05.

HBP was dramatically increased in DN sepsis group(p<0.05), similar elevation degrees also showed in CRP and PCT (p<0.05). ESR and NE% in DN sepsis group were higher than the other two groups (all p<0.05). In DN group, HBP exhibited an upward trend than T2DM group(p<0.05). The results were presented in Table-II.

**Table-II:** Inflammatory indicators of patients in three groups.

Inflammatory indicators	T2DM group	DN group	DN sepsis group	F	p*
HBP (ng/mL)	8.61±5.19	22.28±26.97	286.00±120.20	193.27	<0.001
WBC (×10^9^/L)	6.29±0.78	6.78±1.01	7.62±2.81	5.72	0.004
NE (%)	56.54±7.51	59.92±6.54	67.48±15.53	11.19	<0.001
CRP (mg/L)	1.97±1.12	2.73±1.61	43.68±21.91	141.14	<0.001
PCT (ng/mL)	0.12±0.12	0.11±0.07	1.71±1.99	25.53	<0.001
ESR (mm/h)	17.21±1.34	16.64±1.14	27.20±5.21	140.49	<0.001

* One-way ANOVA, p<0.05.

**Fig.1:**
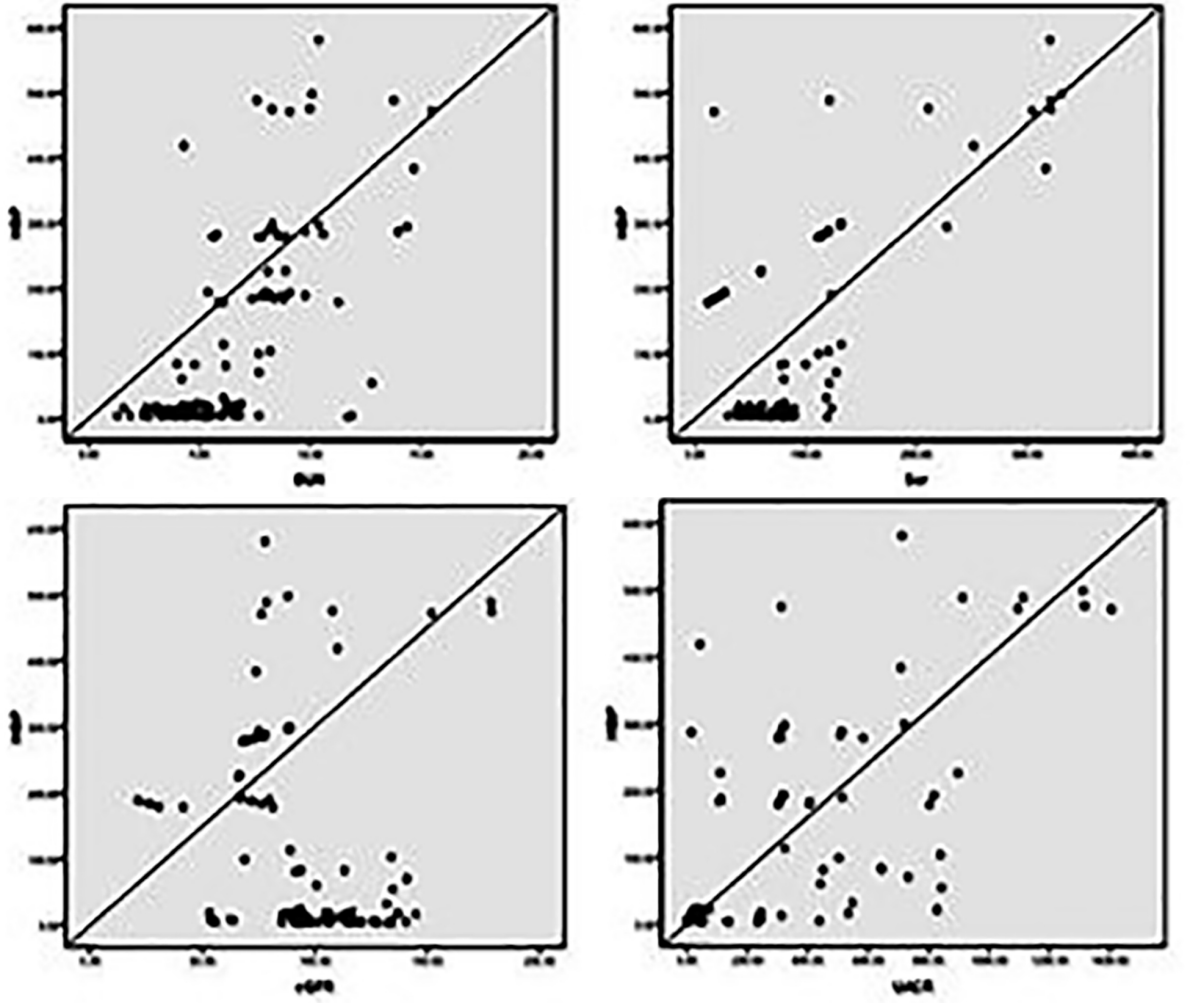
Correlations between the plasma HBP and renal function indicators including: BUN, Scr, eGFR, UACR in DN patients with/without sepsis.

**Fig.2:**
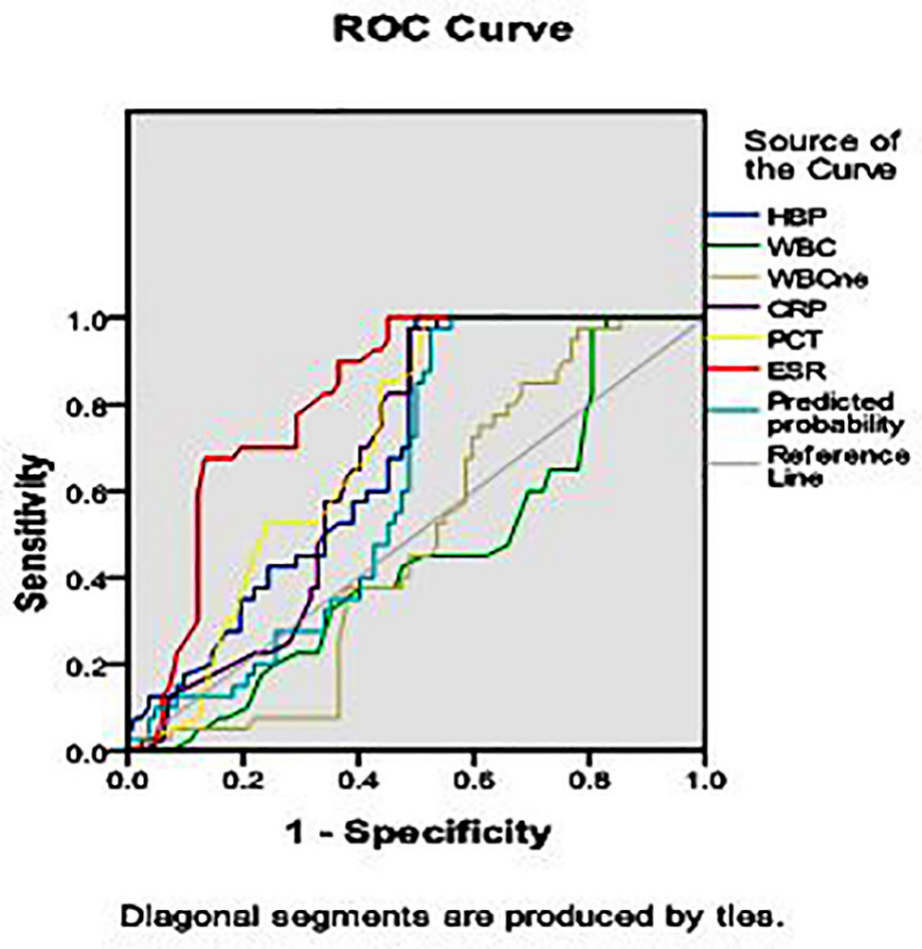
ROC curve of inflammatory indicators for early diagnosis of sepsis in DN patients.

Table-III and Fig.1 suggested the inflammatory indicator HBP was positively correlated with the renal function indicators including BUN, Scr and UACR, negatively correlated with eGFR. The results in Table-IV and Fig.2 manifested that the area under the curve (AUC) of HBP, WBC, NE%, CRP, PCT, ESR were 0.691, 0.452, 0.490, 0.677, 0.709 and 0.818 respectively in the diagnosis of sepsis in DN patients. The combination of all inflammatory indicator results in an AUC of 0.622.

**Table-III:** Correlations between inflammatory and renal function, glucose metabolism and indicators in DN patients with/without sepsis.

Clinical indicators	HBP		CRP		PCT	
	r	p	r	p	r	p*
FBG	0.230	0.011	0. 225	0.013	0.068	0.453
HbA1c	0.249	0.006	0.109	0.233	0.056	0.540
BUN	0.675	<0.001	0.546	<0.001	0.340	<0.001
Scr	0.659	<0.001	0.312	<0.001	0.347	<0.001
eGFR	-0.187	0.039	-354	<0.001	-0.163	0.072
UACR	0.707	<0.001	0.669	<0.001	0.261	0.004

* Pearson correlation analysis, p< 0.05.

**Table-IV:** Diagnostic values of inflammatory indicators in DN comorbid with sepsis patients were performed by ROC curve analysis.

Inflammatory indicators	AUC	Youden Index	Cut-off Value	Sensitivity (%)	Specificity (%)	95% CI	p*
HBP (ng/mL)	0.691	0.500	108.50	1.000	0.500	0.599-0.783	0.001
WBC (×10^9^/L)	0.452	0.171	8.75	1.000	0.171	0.346-0.557	0.386
NE (%)	0.490	0.195	71.45	0.975	0.220	0.389-0.592	0.864
CRP (mg/L)	0.677	0.487	5.50	0.975	0.512	0.584-0.770	0.002
PCT (ng/mL)	0.709	0.488	0.295	1.00	0488	0.621-0.798	<0.001
ESR (mm/h)	0.818	0.549	19.10	1.00	0.549	0.744-0.892	<0.001
Combination	0.622	0.524	/	0.975	0.476	0.524-0.720	0.030

* P< 0.05.

## DISCUSSION

In our study, we detected inflammatory markers within half an hour and one hour after clinical diagnosis of sepsis in DN patients. The levels of HBP, CRP and PCT were significantly higher in DN patients than in non-sepsis patients, indicating that these indicators are sensitive markers for the diagnosis of sepsis in DN patients. We further investigated the trend of changes in inflammatory markers in DN patients without sepsis. Interestingly, only DN patients showed a statistically significant increase in HBP compared to T2DM patients, but the total serum HBP level in the DN group remained within the normal range. In addition, plasma HBP is positively correlated with BUN, Scr and UACR and negatively correlated with eGFR in DN patients, which can reflect the progression of renal injury in DN patients, especially when comorbid with sepsis. Fisher J et al. found that higher plasma HBP concentrations in patients with septic shock can lead to more severe kidney damage.^9^ Our research results also indicate that changes in plasma HBP may be related to the pathological generation and progression of kidney injury. ROC analysis showed that HBP provides better ACU than other inflammatory markers such as WBC or NE%, indicating that HBP has higher sensitivity and specificity in diagnosing sepsis from DN patients. DN with high mortality and serious threat to patients’ lives.^10^-^13^ The potential pathogenesis of DN is complex, consisting of a combination of inflammatory response, oxidative stress, abnormal activation of glucose metabolism pathways and renal hemodynamic imbalance under hyperglycemic conditions.^14^ Patients with diabetes are highly susceptible to bacterial infectious diseases. Compared with age matched non diabetes patients, patients with diabetes have a significantly higher risk of sepsis.^15^,^16^ Therefore, early identification, diagnosis and treatment of sepsis in DN patients can not only limit the progression of sepsis and even septic shock, but also reduce the dual impact of high blood sugar and sepsis on the kidneys, lower the risk of kidney failure and save the lives of patients.

Sepsis is accompanied by life-threatening multiple organ failure in most cases. Due to the high incidence rate and mortality, sepsis is still the main cause of death worldwide.^17^ HBP is a pro-inflammatory protein that is rapidly released in large quantities by neutrophils when the body is attacked by bacteria. Research has shown that the HBP levels in sepsis patients are significantly higher than those in patients with local infections and HBP levels can directly reflect the severity of sepsis.^18^,^19^ In addition, elevated HBP can amplify the inflammatory response in the body by inducing inflammatory cells, activating endothelial function, increasing vascular permeability and exacerbating inflammation related microcirculatory disorders.^20^

### Limitations

There are still some shortcomings in this study. Fewer samples were included in this study, and the corresponding results need to be verified in a more large-scale sample size. In response to this, the sample size will be further increased in future studies to validate the results of this study.

## CONCLUSIONS

The plasma HBP in DN with sepsis is significantly increased, which is closely related to diabetes kidney damage, may reflect the progress of renal dysfunction under severe infection and may improve the early diagnosis efficiency of DN with sepsis.

### Authors’ Contributions:

**LH:** Participated in data analysis and wrote the original manuscript.

**SW:** Designed research and reviewed manuscript.

**ZW:** Screened the eligible study participants.

All authors have approved the final version and are accountable for the integrity of the study.

## References

[1] Yang J, Liu Z (2022). Mechanistic Pathogenesis of Endothelial Dysfunction in Diabetic Nephropathy and Retinopathy. Front Endocrinol (Lausanne).

[2] Młynarska E, Czarnik W, Dzieża N, Jędraszak W, Majchrowicz G, Prusinowski F (2025). Type-II Diabetes Mellitus: New Pathogenetic Mechanisms, Treatment and the Most Important Complications. Int J Mol Sci.

[3] Salomão R, Ferreira BL, Salomão MC, Santos SS, Azevedo LCP, Brunialti MKC (2019). Sepsis: evolving concepts and challenges. Braz J Med Biol Res.

[4] Samuelsson L, Tydén J, Herwald H, Hultin M, Walldén J, Steinvall I (2019). Renal clearance of heparin-binding protein and elimination during renal replacement therapy: Studies in ICU patients and healthy volunteers. PLoS One.

[5] American Diabetes Association (2021). 11. Microvascular Complications and Foot Care: Standards of Medical Care in Diabetes-2021. Diabetes Care.

[6] American Diabetes Association (2021). 2. Classification and Diagnosis of Diabetes: Standards of Medical Care in Diabetes-2021. Diabetes Care.

[7] Singer M, Deutschman CS, Seymour CW, Shankar-Hari M, Annane D, Bauer M (2016). The Third International Consensus Definitions for Sepsis and Septic Shock (Sepsis-3). JAMA.

[8] Jia Y, Guan M, Zheng Z, Zhang Q, Tang C, Xu W (2016). miRNAs in Urine Extracellular Vesicles as Predictors of Early-Stage Diabetic Nephropathy. J Diabetes Res. 2016.

[9] Fisher J, Linder A, Bentzer P, Boyd J, Kong HJ, Lee T (2018). Is Heparin-Binding Protein Inhibition a Mechanism of Albumin's Efficacy in Human Septic Shock?. Crit Care Med.

[10] Khan NU, Lin J, Liu X, Li H, Lu W, Zhong Z (2020). Insights into predicting diabetic nephropathy using urinary biomarkers. Biochim Biophys Acta Proteins Proteom.

[11] Han L, Wang S, Ma J, Song N, Wang Z, Yao M (2023). Changes of Serum Bone Metabolism Indexes and Ultrasonic Bone Mineral Density in Patients with Diabetic Nephropathy at Different Stages and their effects on Diabetic Renal Microvascular Complications. Pak J Med Sci.

[12] Liu J, Zhang J, Hou MH, Du WX (2022). Clinical efficacy of linagliptin combined with irbesartan in patients with diabetic nephropathy. Pak J Med Sci.

[13] Efe FK (2021). The association between monocyte HDL ratio and albuminuria in diabetic nephropathy. Pak J Med Sci.

[14] Huang M, Cai S, Su J (2019). The Pathogenesis of Sepsis and Potential Therapeutic Targets. Int J Mol Sci.

[15] Costantini E, Carlin M, Porta M, Brizzi MF (2021). Type-II diabetes mellitus and sepsis: state of the art, certainties and missing evidence. Acta Diabetol.

[16] Zohar Y, Zilberman Itskovich S, Koren S, Zaidenstein R, Marchaim D, Koren R (2021). The association of diabetes and hyperglycemia with sepsis outcomes: a population-based cohort analysis. Intern Emerg Med.

[17] Purcarea A, Sovaila S (2020). Sepsis, a 2020 review for the internist. Rom J Intern Med.

[18] Tian R, Chen X, Yang C, Teng J, Qu H, Liu HL (2021). Serum Heparin-Binding Protein as a Potential Biomarker to Distinguish Adult-Onset Still's Disease from Sepsis. Front Immunol.

[19] Wang Z, Chang B, Zhang Y, Chen J, Xie F, Xiang Y (2022). Clinical value of serum sTREM-1 and HBP levels in combination with traditional inflammatory markers in diagnosing hospital-acquired pneumonia in elderly. BMC Infect Dis.

[20] Feng L, Liu S, Wang J, Gao Y, Xie F, Gong J (2024). The performance of a combination of heparin-binding protein with other biomarkers for sepsis diagnosis: an observational cohort study. BMC Infect Dis.

